# TLR4/NF-*κ*B Signaling Contributes to Chronic Unpredictable Mild Stress-Induced Atherosclerosis in ApoE^-/-^ Mice

**DOI:** 10.1371/journal.pone.0123685

**Published:** 2015-04-10

**Authors:** Ya Ling Tang, Jian Hong Jiang, Shuang Wang, Zhu Liu, Xiao Qing Tang, Juan Peng, Yong-Zong Yang, Hong-Feng Gu

**Affiliations:** 1 Key Lab for Arteriosclerology of Hunan Province, Institute of Cardiovascular Disease, University of South China, Hengyang, 421001, People's Republic of China; 2 Chuanshan College, University of South China, Hengyang, 421001, People’s Republic of China; 3 Department of Physiology & Institute of Neuroscience, University of South China, Hengyang, 421001, People’s Republic of China; Max-Delbrück Center for Molecular Medicine (MDC), GERMANY

## Abstract

**Objective:**

Chronic stress is an important risk factor for atherosclerotic diseases. Our previous studies have shown that chronic unpredictable mild stress (CUMS) accelerates atherosclerosis and up-regulates TLR4/NF-*κ*B expression in apoE^-/-^ mice. However, TLR4/NF-*κ*B signaling whether directly contributes to the development of atherosclerosis in CUMS mice is unclear. We hypothesized that the interference of TLR4/NF-*κ*B can ameliorate CUMS-induced inflammation and atherosclerosis in apoE^-/-^ mice.

**Methods:**

ApoE^-/-^ mice were exposed to 12 weeks CUMS. Ad-siRNA TLR4 was given by tail vein injection (10 μl/mouse, every 5 days), and PDTC (an inhibitor of NF-*κ*B) was given by intraperitoneal injection (60 mg/kg, once a day). Plasma corticosterone concentrations were determined by solid-phase ^125^I radioimmunoassay. Atherosclerosis lesions in aortic sinuses were evaluated and quantified by IMAGEPRO PLUS. Western blotting was used to detect the expression of TLR4, NF-*κ*B, and IL-1β in aortas of the mice. Plasma lipid profiles, IL-1β, TNF-α, and MCP-1 were measured by ELISA.

**Results:**

Our results indicated that CUMS apoE^-/-^ mice treatment with siRNA TLR4 significantly decreased atherosclerosis and down-regulated TLR4, NF-*κ*B, and inflammatory cytokines. PDTC also remarkably reduced atherosclerosis and the levels of IL-1β, TNF-α and MCP-1 in plasma. However, Treatment with siRNA TLR4 or PDTC had no effect on plasma corticosterone levels, and lipid profiles.

**Conclusions:**

TLR4/NF-*κ*B pathway may participate in CUMS-induced atherosclerosis through activation of proinflammatory cytokines in apoE^-/-^ mice. Our data may provide a new potential therapeutic target for prevention of CUMS -induced atherosclerosis.

## Introduction

Atherosclerosis is an inflammatory disease of the arterial wall [1, 2]. Various risk factors, such as smoking, dyslipidemia, hypercholesterolemia, and obesity, contribute to this disease [3]. However, the absence of such "traditional" risk factors does not completely protect from atherosclerotic disease, suggesting that additional factors implicated in the initiation and progression of atherosclerosis. A considerable amount of epidemiological and experimental evidence have shown that chronic stress, especially chronic psychological stress, may play important roles in the development of atherosclerosis [4–12]. Accumulating clinical data has also indicated that psychosocial stress is an independent risk factor in the development of atherosclerosis [13–14]. More evidence suggested that intervention for stress reduction may be associated with a decreased carotid atherosclerosis in terms of intima-media thickness [15]. Although the importance of chronic stress in atherosclerosis has gained wide spread acceptance, little is known about the precise signaling pathways.

Chronic inflammation may be a potential mechanism mediating chronic stress induced atherosclerosis diseases. It has been reported that chronic stress results in the elevation of plasma levels of CRP and interleukin-6 (IL-6), suggesting that repeated exposure to stress may induce inflammatory response [16–18]. However, the mechanisms of chronic stress associated with the inflammation and atherosclerosis are not soundly established. Among the various signal transduction pathways implicated in proatherogenic inflammation, the Toll-like receptor 4 (TLR4)/nuclear factor kappa B (NF-*κ*B) pathway has recently aroused more attention due to its important roles in atherosclerosis [19–21]. TLR4 is expressed in macrophages, vascular endothelial and smoother muscle cells [22]. Once activated by its ligands, TLR4 can activate NF-*κ*B signaling pathway linked to the transcription of many proinflammatory genes. Michelsen et al. reported that the deficiency of TLR4 dramatically reduced aortic atherosclerotic lesion areas and circulating levels of proinflammatory cytokines in apoE^-/-^ mice [20]. Resent studies also showed that TLR4 might play important roles in chronic stress-induced inflammation response [23–25]. Wang et al. reported that TLR4/NF-*κ*B pathway was involved in the myocardial injury after chronic stress [26]. Interestingly, our previous study also showed that chronic unpredictable mild stress (CUMS) promoted the development of aortic atherosclerosis in apoE^-/-^ mice, which may be related to activation of TLR4 and NF-*κ*B in the aorta [11]. In addition, mediators isolated after chronic stress have been identified as ligands of TLR4 [11, 25, 27]. However, whether this signaling pathway directly contributes to stress-induced atherogenesis is still not fully clear.

Given that the causal relationship between vascular inflammation and atherogenesis, we hypothesized that TLR4/NF-*κ*B pathway might be directly involved in CUMS-induced atherosclerosis. To test the hypothesis, we used a CUMS model in apoE^-/-^ mice to investigate the effects of TLR4 small interfering RNA (siRNA) and a specific NF-*κ*B antagonist pyrrolidine dithiocarbamate (PDTC) on stress-induced inflammation and atherosclerosis. We expected to illuminate the roles of TLR4/NF-*κ*B pathway in chronic stress-induced atherosclerosis. Our results suggested that blocking TLR4/NF-*κ*B pathway significantly attenuated the aortic atherosclerotic lesions and inflammation in CUMS apoE^-/-^ mice.

## Materials and Methods

### Ethics statement

This study was carried out in strict accordance with the recommendations in the Guide for the Care and Use of Laboratory Animals of the National Institutes of Health. The protocol was approved by the Committee on the Ethics of Animal Experiments of the University of South China (Permit Number: 20140064). All surgery was performed under sodium pentobarbital anesthesia, and all efforts were made to minimize suffering.

### Preparation of recombinant adenovirus expressing small interference RNA of TLR4 (Ad-TLR4 siRNA)

Screening, preparation and testing sequence, and the knock-down efficiency SiRNA-TLR4 in 293A cells were performed as described in detail previously by us [28]. Briefly, the target gene TLP4 siRNA was designed and synthesized by Shanjing Company (Shanghai, China) with the following sequences. Sense strand: 5'-CGCGTGTATTACCTACCAATGCATGTTGATATCCGCATGCATTGGTAGGTAATATTTTTTCCAAA-3' and an antisense strand: 5'-AGCTTTTGGAAAAAAT ATTACCTACCAATGCATGCGGATATCAACATGCATTGGTAGGTAATACaA-3'. BamH I and Hind III sites were included at the 5’ and 3’ ends, respectively. After pShuttleH1was digested by Bgl II and Hind III, the target gene siRNA was cloned into pShuttleH1 and named as pShuttleH1-siRNA. Then pShuttleH1-siRNA was linearized by Pme I digestion and transformed into Escherichia coli containing backbone plasmid pAdEasy-1 by electroporation. The correct clone was identified and sequenced by Shanjing Company (Shanghai, China). Then linearized recombinant plasmid was transfected into HEK 293A cells. To test whether wild-type virus emerged during the amplification, the E1 region of adenovirus was detected by RT-PCR. The titer of concentrated virus was evaluated by hole-by-dilution titer assay.

### Animals

Male apoE^-/-^ mice weighing 16.5 ± 0.5 g (4 weeks old) were obtained from the Laboratory Animal Center of Peking University, Beijing, China. After arrival, the mice were housed 5 per small polycarbonate cage (8 × 13.5 × 8.1 cm) in a temperature and humidity-controlled environment and maintained in a 12-hour dark/light cycle (lights on at 7:00 AM) room. The mice were allowed to habituate to laboratory conditions for at 1 week prior to use.

### Groups and drug treatments

The mice were randomly assigned to the following 6 groups (n = 15/group): control group, CUMS group, CUMS + Ad-TLR4 siRNA (CUMS + siRNA) group, CUMS + empty vector group, CUMS + PDTC group, and vehicle (sterile PBS) + CUMS group. The apoE^-/-^ mice were exposed to CUMS with or without drug treatments for 12 weeks. Ad-TLR4 siRNA was given by tail vein injection (10 μl/mouse, the virus with titer of 3.4 × 10^11^ infectious units per ml, every 5 days), and PDTC (an inhibitor of NF-*κ*B) was given by intraperitoneal injection (100 mg/kg, once a day) for consecutive 12 weeks. The doses of siRNA and PDTC were chosen based on previous studies [28, 29]. Drugs and vehicles were administered at 30 min prior to stress exposure. At 5 weeks, all mice were fed an atherogenic diet (containing 5% fat and 1.0% cholesterol) during the experimental period of 12 weeks. The control mice were housed separate room and had no contact with the stressed animals. Food and water were available ad libitum.

### Chronic unpredictable mild stress procedures

The mice were subject to the CUMS as described in detail previously by us and Ipek Yalcin et al. [11,30]. Briefly, the stress group was subjected to the CUMS for 12 weeks. Each week of stress regime consisted of the following stressors: two periods (7 and 17 hours) of continuous overnight illumination; two periods (7 and 17 hours) of 45 degrees cage tile, one 17 hours period in a soiled cage (100 ml water in sawdust bedding), two periods (9 and 15 hours) of intermittent sound (a tone of 80 dB), three periods (7, 9 and 17 hours) of low-intensity stroboscopic illumination (150 flashes/min), and two periods of exposure to rat odour (removal of the cage containing the experimental mice into the procedure room and placing the experimental mice into cages in which rats had been held). To avoid habituation, stressors were assigned in random order while assuring that each animal were received the same numbers of stressors at the end of the experiments. Food consumption was assessed by monitoring the food intake daily (the weight of food given in the morning minus the leftover next morning) during the last week of treatment. All the mice were euthanized at the end of week 12 and body weight was measured immediately after animal euthanasia.

### Assessment of the plasma corticosterone concentration

After 12 weeks stress, all mice were sacrificed and blood samples were obtained by heart puncture. The plasma corticosterone concentrations were determined by solid-phase ^125^I radioimmunoassay using a commercially available reagent kit (Diagnostic Products, Los Angeles, CA) as described in our previous study [11, 31].

### Assessment of atherosclerosis in aortas and aortic sinuses

Tissue preparation and quantification of atherosclerosis in aortas and aortic sinuses were performed as previously described [11]. Briefly, whole aortas from the aortic arch to the iliac bifurcation were removed and cleaned of adventitia, then opened longitudinally and stained for lipids with Soudan IV. To determine cross-sectional lesion area, hearts were embedded in OCT compound (Tissue Tek, Sakura, Torrance, CA), frozen on dry ice, and then stored at -70°C until sectioning. Serial sections 6 μm thick were collected on slides and stained with oil red O (Sigma, USA). Cross sections of the aortic sinus and aortic valve were stained with oil red O and counterstained with Gill III hematoxylin (Sigma, USA). Atherosclerotic lesion areas in the aortic sinus were measured by a blinded observer and quantified with IMAGEPRO PLUS. Data are expressed as the average lesion size per section or the percent of the total aortic surface area in the arch that stained with Oil red O. For each animal, the average of 12 sections was determined.

### Assessment of plasma lipid profile

Total cholesterol (TC), triglyceride (TG), low-density cholesterol (LDL-c) and high-density lipoprotein cholesterol (HDL-c) concentrations in plasma were measured using enzymatic assays kits according to manufacturer’s instructions.

### Western blot analysis

To examine protein expressions of TLR4, NF-*κ*B (p65), I*κ*B-α and IL-1β in aortic tissues, aortas were removed and cleaned of adventitia from experimental mice. These tissues were washed with PBS and lysates were prepared by lysis buffer. Nuclei and cytoplasmic debris were pelleted at 14,000 × g for 20 min, and supernatants were collected. The protein content was determined by Bradford assay. Equal amounts of proteins (30 μg protein/lane) were electrophoresed using 10% sodium dodecyl sulfate polyacrylamide gels in a Tris/HCl buffer system, followed by electrophoretic transfer to a polyvinylidene difluoride microporous membrane (BioRad, Hercules, PA, USA). Subsequently, the membranes were incubated with the appropriate primary antibodies overnight at 4°C: rabbit anti-mouse β-actin (abcam, ab6276, 1:5000), rabbit anti-mouse TLR4 antibody (abcam, ab47093, 1:1000), rabbit anti-mouse I*κ*B-α (abcam, ab32518, 1:1500) rabbit anti-mouse NF-*κ*B p65 (cell signaling technology, 3037, 1:1000) and IL-1β (abcam, ab106035, 1:1000). Following 3 washes with TBST buffer, immunodetection was accomplished using appropriate horseradish peroxidase-linked secondary antibodies (KPL, 074–1516) and enhanced chemiluminescence system. Protein band densities were quantified by Image-J software (NIH).

### Assessment of plasma LPS, LBP, IL-1β, TNF-α, and MCP-1 levels in plasma

Plasma lipopolysaccharide (LPS) and Lipopolysaccharide binding protein (LBP) levels were determined using commercially available kits following the manufacturer’s instructions (Hycult Biotech, The Netherlands). Plasma LPS was measured using a chromogenic endpoint assay at 450 nm in a spectrophotometer (Molecular Devices). LBP was measured at 450 nm in a spectrophotometer (Molecular Devices). The results are expressed as ng/ml of plasma.

Plasma levels of IL-1β, TNF-α, and MCP-1 were measured by enzyme linked immunosorbent assay (ELISA) kits (Minneapolis, MN, USA) according to the manufacturer instructions.

### Statistical Analysis

All data were presented as mean ± SEM. The data were subjected to one-way analysis of variance (ANOVA) followed by Student-Newman-Keuls (SNK-q) multiple range test for comparison of mean values (PRISM software). A value of *P* < 0.05 was considered statistically significant.

## Results

### TLR4 siRNA and PDTC treatments do not influence food intake and body weight in CUMS apoE^-/-^ mice

**Fig 1:**
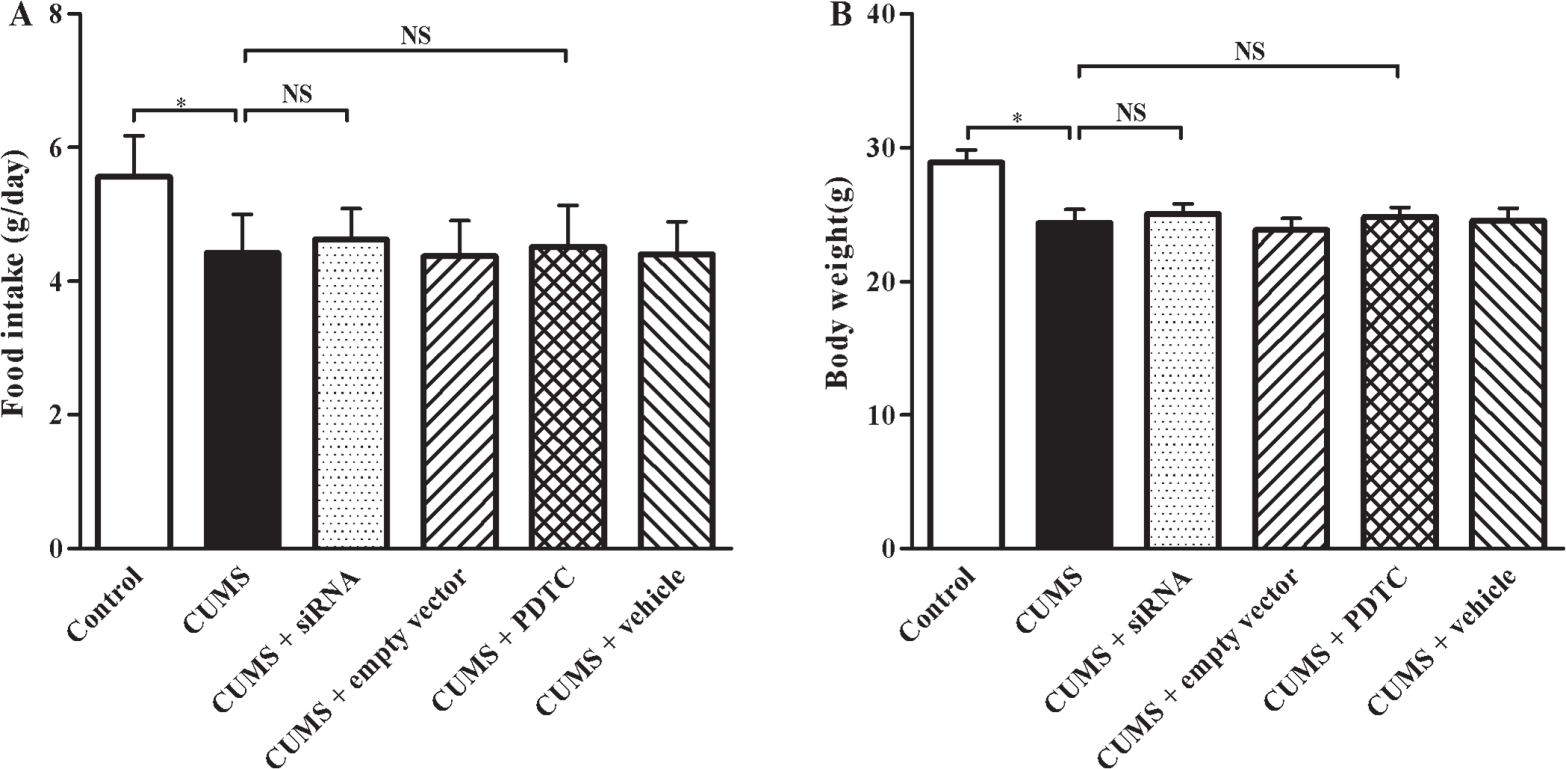
Effect of TLR4 siRNA and PDTC treatment on food intake and body weight in CUMS apoE^-/-^ mice. (A) Food intake and (B) Body weight were determined at week 12 of experimental procedure. Food intake and body weight in CUMS mice significantly decreased compared with that in control ones, respectively. There were no significant differences in food consumption and body weight among CUMS + siRNA, CUMS + empty vector group, CUMS + PDTC, CUMS + vehicle group, and CUMS group. The agents (siRNA and PDTC) were given to mice 30 min before the stress exposure. Data are shown as mean ± SEM (n = 15 for each group). NS, no significant difference. *P < 0.05.

After 12 weeks stress, the average food intake (Fig 1A, *P* < 0.05) and body weight (Fig 1B, *P* < 0.05) were significantly decreased in CUMS group compared with those in control group, respectively. However, there were no significant differences in food consumption and body weight among CUMS + siRNA, CUMS + empty vector group, CUMS + PDTC, CUMS + vehicle group, and CUMS group (*P* > 0.05).

### TLR4 siRNA and PDTC treatments do not influence plasma corticosterone levels in CUMS apoE^-/-^ mice

**Fig 2:**
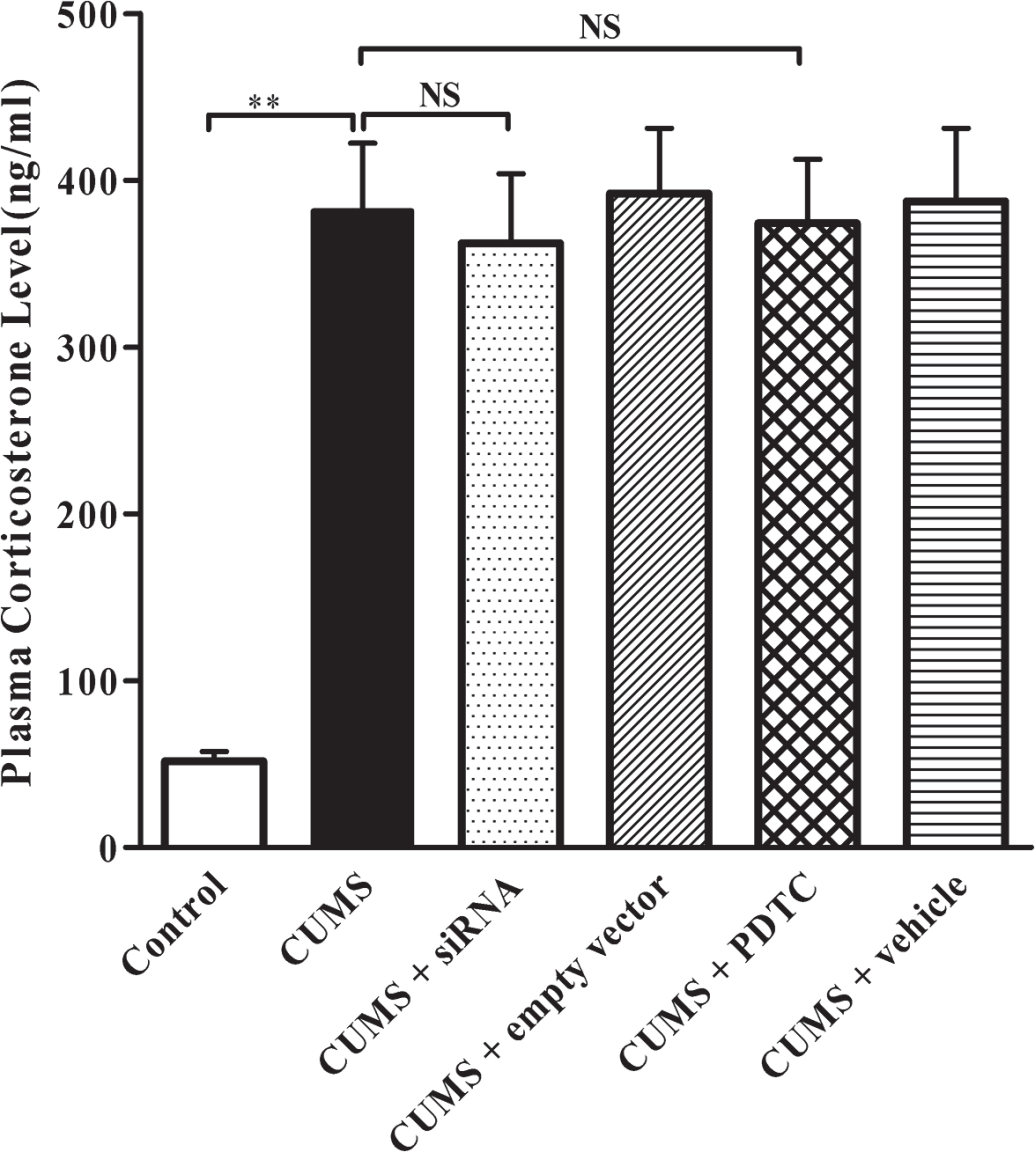
Effect of TLR4 siRNA and PDTC treatment on the plasma corticosterone levels in apoE^-/-^ mice. Control, control group; CUMS, chronic unpredictable mild stress group; CUMS + siRNA, siRNA administration followed by CUMS group; CUMS + empty vector, empty vector treatment followed by CUMS group; CUMS + PDTC, administration with PDTC followed by CUMS group; CUMS + Vehicle, vehicle treatment followed by CUMS group. The agents (siRNA and PDTC) were given to mice 30 min before the stress exposure. Data are shown as mean ± SEM (n = 15 for each group). NS, no significant difference. **P < 0.01.

The hypothalamic–pituitary–adrenal axis is a major effector of responses to systemic stress. We therefore measured the plasma corticosterone levels to ensure that CUMS paradigms induced sufficient stress. Similar to our previous study [11], after 12 weeks of CUMS, plasma corticosterone concentration was significantly higher in the CUMS group than that in the control group (Fig 2, *P* < 0.05). However, the plasma corticosterone concentrations were not significantly changed in CUMS + siRNA, CUMS + empty vector group, CUMS + PDTC and CUMS + vehicle group, compared with CUMS group (Fig 2, *P* > 0.05), respectively.

### Inhibition of TLR4/NF-*κ*B significantly reduces the extent of aortic atherosclerosis in CUMS apoE^-/-^ mice

**Fig 3:**
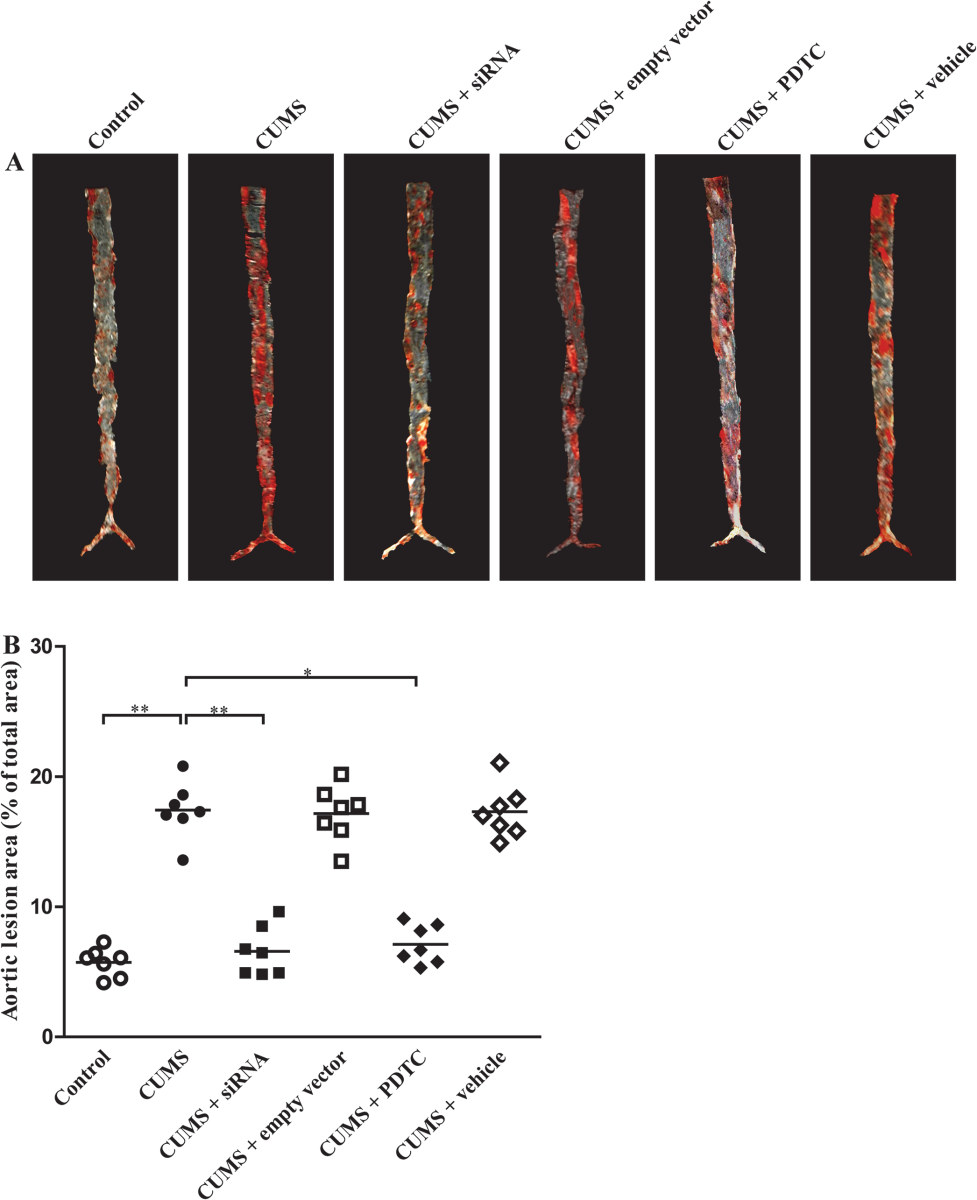
Effect of TLR4 siRNA and PDTC treatment on atherosclerotic lesions in aortas of CUMS apoE^-/-^ mice. (A) Soudan IV staining of en face aortas from 6 groups. (B) Percentage of atherosclerotic lesion areas in the en face aortas of the apoE^-/-^ mice. The lesion areas in CUMS + siRNA group, and CUMS + PDTC group were significantly decreased than that of CUMS groups, respectively. The agents (siRNA and PDTC) were given to mice 30 min before the stress exposure. Data are shown as mean ± SEM (n = 7 for each group). *P < 0.05, **P < 0.01.

To evaluate the effect of TLR4 siRNA and PDTC on the extent of aortic atherosclerosis, we measured the total lesion area by using en face preparation of the aorta and Soudan IV staining for lipids. As shown in Fig 3, CUMS treatment significantly increased both atherosclerotic plaque size (Fig 3A) and plaque-to-surface ratio (Fig 3B) in apoE^-/-^ mice, compared to the control ones, respectively. Quantitative analysis revealed a remarkable reduction in the extent of atherosclerosis both in CUMS + siRNA group and CUMS + PDTC group mice, as compared with that in control ones.

### Inhibition of TLR4/NF-*κ*B significantly reduces the extent of aortic sinus atherosclerosis in CUMS apoE^-/-^ mice

**Fig 4:**
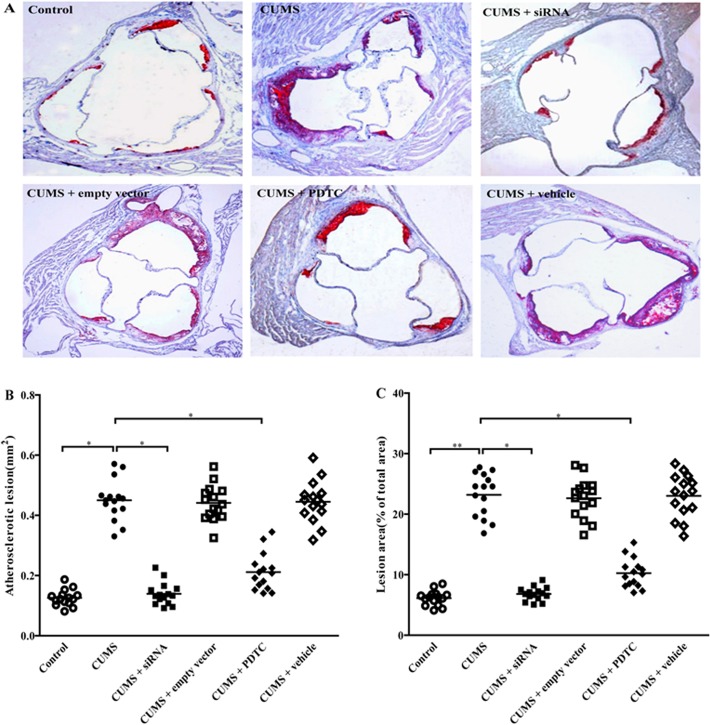
Effect of TLR4 siRNA and PDTC treatment on atherosclerotic lesions in aortic sinuses of CUMS apoE^-/-^ mice. Atherosclerotic lesions in aortic sinuses of apoE^-/-^ mice subjected to CUMS or control for 12 weeks. (A) 6 *μ*m frozen sections of the aortic sinuses were stained for lipid deposition with oil red O (Original magnification × 100). CUMS markedly increased atherosclerosis in aortic sinuses of apoE^-/-^ mice. (B) Quantification of atherosclerotic lesions in aortic sinuses in apoE^-/-^ mice. The atherosclerotic lesions in CUMS + siRNA group, and CUMS + PDTC group were significantly lowered than that of CUMS group, respectively. (C) Percentage of atherosclerotic lesion areas in entire aorta in apoE^-/-^ mice. The lesion area in CUMS + siRNA group, and CUMS + PDTC group were significantly decreased than that of CUMS groups, respectively. The agents (siRNA and PDTC) were given to mice 30 min before the stress exposure. Data are shown as mean ± SEM (n = 15 for each group). *P < 0.05, **P < 0.01.

To further investigate the effects of TLR4 siRNA and PDTC treatments on the atherosclerosis in CUMS apoE^-/-^ mice, serial sections of the aortic sinus and aortic valve were stained with oil red O and atherosclerotic lesions were measured. As shown in Fig 4, after 12 weeks stress, the atherosclerotic lesions in aortic sinuses were significantly larger in CUMS mice, compared to the control mice, denoting a profound exacerbation of atherosclerosis by CUMS (Fig 4B, *P* < 0.05). As expected, the lesions in CUMS + siRNA group, and CUMS + PDTC group were significantly reduced compared with CUMS group, respectively (Fig 4B, both *P* < 0.05), indicating that inhibition of TLR4/NF-*κ*B obviously attenuated CUMS-induced atherosclerosis. There were no significant differences in atherosclerotic lesions among CUMS + empty vector group, CUMS + vehicle group, and CUMS group (Fig 4B, *P* > 0.05).

Similar results were seen in Fig 4C, the percentage changes of aortic atherosclerotic lesions in entire aortic surface areas (% aorta covered by plaque). In CUMS + siRNA group and CUMS + PDTC group were significantly decreased, compared with CUMS group (Fig 4C, both *P* < 0.05). These results suggested that TLR4/NF-*κ*B was involved in CUMS-promoted atherosclerosis in apoE^-/-^ mice. No differences were observed among CUMS + empty vector group, CUMS + vehicle group, and CUMS group (Fig 4C, *P* > 0.05).

### siRNA TLR4 and PDTC treatment do not significantly influence plasma lipid profile in CUMS apoE^-/-^ mice

To evaluate whether siRNA TLR4 and PDTC treatments affect lipid profile, plasma lipid concentrations were measured after 12 weeks stressor exposure. As shown in Table 1, mean plasma TC, TG, and LDL-c levels were significantly elevated in CUMS mice compared with control mice (*P* < 0.05). However, plasma HDL-c content in CUMS mice was remarkably lower than that in control mice (*P* < 0.05). Interestingly, concentrations of plasma TC, TG, HDL-c, and LDL-c were not significantly different in CUMS + siRNA group and CUMS + PDTC group compared with CUMS group. These data indicated that siRNA TLR4 and PDTC treatments did not obviously influence lipid metabolism in CUMS mice.

**Table 1:** Effect of TLR4 siRNA and PDTC treatment on the plasma lipid profile of CUMS apoE^-/-^ mice.

Group	TC (mg/dL)	TG (mg/dL)	HDLc (mg/dL)	LDLc (mg/dL)
**Control**	1207.8 ±103.7	185.2 ± 18.5	263.8 ± 21.8	775.2 ± 74.6
**CUMS**	1612.6 ± 124.1*	285.7 ± 24.7*	192.3 ± 20.1*	1145.7 ±106.1*
**CUMS+siRNA**	1590.7 ± 141.2*	252.1 ± 22.5*	223.6 ± 18.3*	1126.3 ± 90.3*
**CUMS+empty vector**	1638.2 ± 117.6*	281.5 ± 23.6*	211.6 ± 20.5*	1152.7 ±112.4*
**CUMS+PDTC**	1669.6 ± 137.2*	285.3 ± 29.6*	228.1 ± 19.8*	1160.3 ± 99.6*
**CUMS+vehicle**	1623.5 ± 133.8*	271.5 ± 24.2*	219.4 ± 20.7*	1158.2 ± 102.9*

Plasma lipid levels were detected by ELISA. TC: total cholesterol; TG: triglyceride; LDL-c: low-density lipoprotein cholesterol; HDL-c: high-density lipoprotein cholesterol. Data are shown as mean ± SEM (n = 15 for each group),

* *P* < 0.05 compared to the control group.

### Inhibition of TLR4/NF-*κ*B remarkably increases I*κ*B-α and decreases IL-1β expression in aorta of CUMS apoE^-/-^ mice

**Fig 5:**
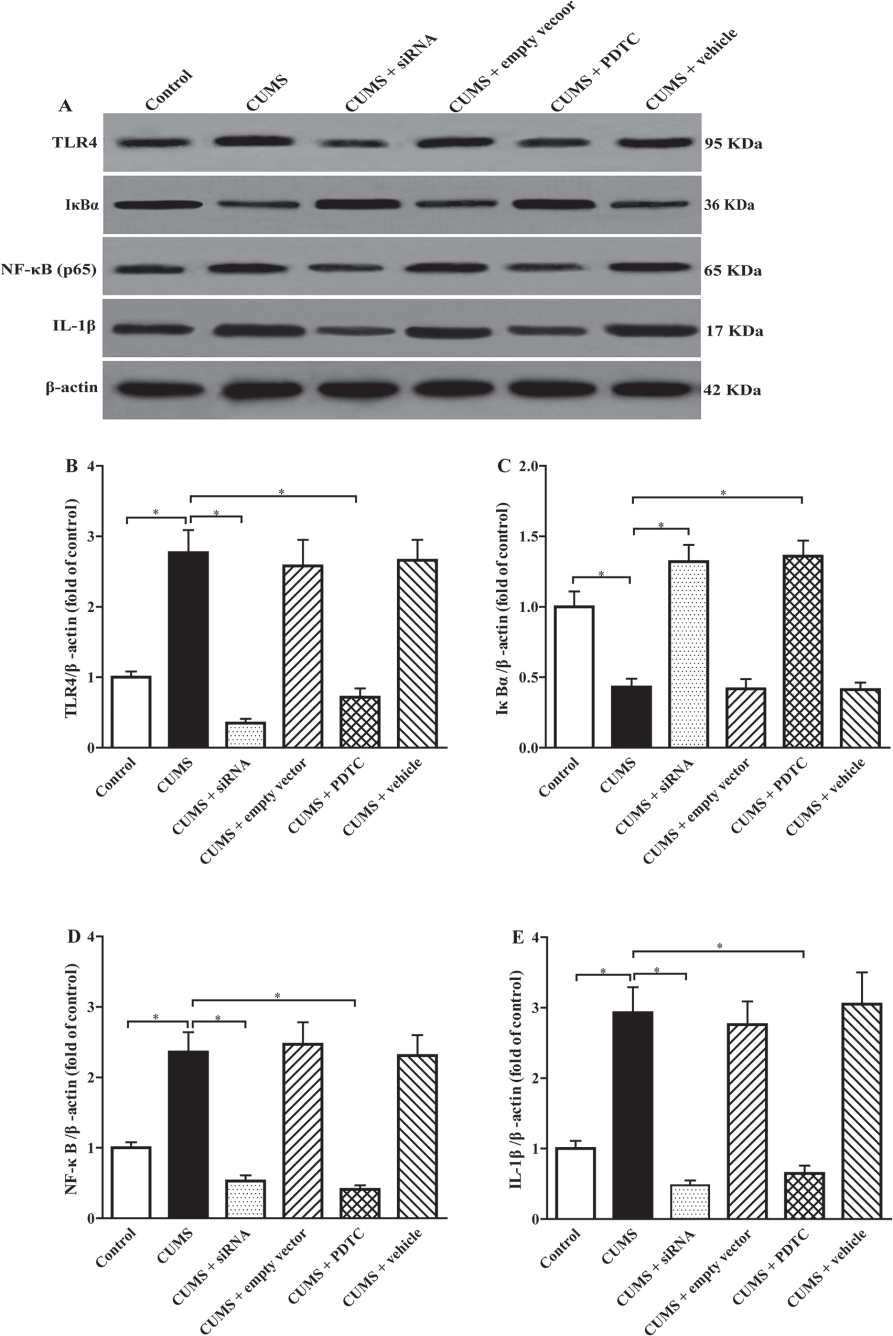
Effect of TLR4 siRNA and PDTC treatment on TLR-4, I*κ*B-α, NF-*κ*B (p65), and IL-1β protein levels in aorta of CUMS apoE^-/-^ mice. (A) TLR4 (95 kDa), I*κ*B-α (36 kDa), NF-*κ*B (65 kDa) and IL-1β (17 kDa) protein levels in aorta were conducted by western blot analysis. 30 μg proteins were loaded in each lane for all experiments. (B), (C), (D), and (E) Densitometry analysis for TLR-4, I*κ*B-α, Intra-nuclear NF-*κ*B (p65), and IL-1β protein levels were performed using three independent experiments, respectively. The agents (siRNA and PDTC) were given to mice 30 min before the stress exposure. Data are mean ± SEM (*n* = 8 per group). *P < 0.05.

Accumulated data have been shown that TLR4, I*κ*B, NF-*κ*B, and IL-1β might be involved in atherosclerotic lesions development. In present study, we investigated the effect of siRNA TLR4 and PDTC treatments on the expression of those proteins in aorta of the apoE^-/-^ mice by Western blotting (Fig 5A). The results showed that CUMS significantly elevated the levels of TLR4 (Fig 5A and 5B), intra-nuclear NF-*κ*B p65 subunit (Fig 5A and 5D), and IL-1β protein (Fig 5A and 5E) and lowered the expression of I*κ*B-α (Fig 5A and 5C) in aorta of apoE^-/-^ mice compared with control group, respectively. Interestingly, siRNA TLR4 treatment obviously suppressed TLR4 (Fig 5A and 5B), intra-nuclear NF-*κ*B p65 (Fig 5A and 5D) and IL-1β (Fig 5A and 5E) expression and increased I*κ*B-α (Fig 5A and 5C) expression in the aorta of CUMS-induced apoE^-/-^ mice, respectively. The same trend was observed in CUMS + PDTC mice (Fig 5A–5E). Our results showed that siRNA TLR4 and PDTC treatments increased expression of I*κ*B-α in the cytoplasm coincides with a reduction of NF-*κ*B p65 in the nucleus, thus reversing CUMS- induced TLR4/NF-*κ*B activation in aortic wall. No significant differences were found in TLR4, IκB-α, NF-*κ*B, and IL-1β protein levels among CUMS + empty vector group, CUMS + vehicle group, and CUMS group (Fig 5A–5E). These results suggested that inhibition of TLR4/NF-*κ*B could attenuate inflammation in aortic wall.

### Inhibition of TLR4/NF-*κ*B reduces plasma proinflammatory cytokines IL-1β, TNF-α, and MCP-1 production in CUMS apoE^-/-^ mice

**Fig 6:**
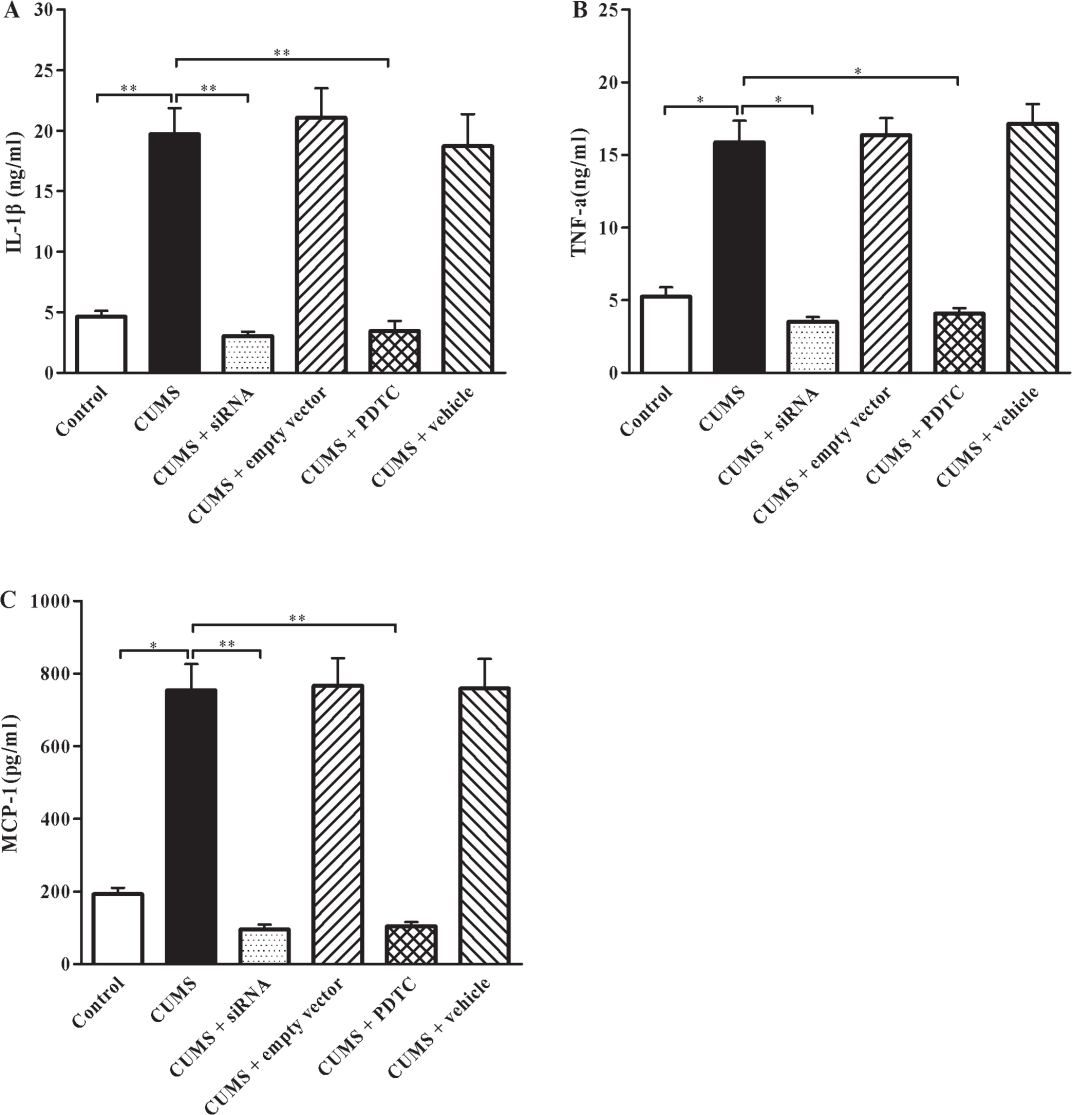
Effect of TLR4 siRNA and PDTC treatment on plasma IL-1β, TNF-α, and MCP-1 levels. (A), (B), and (C) IL-1*β*, TNF-α and MCP-1 levels in the plasma of apoE^-/-^ mice were analyzed by ELISA, respectively. The plasma levels of cytokines IL-1β, TNF-α, and MCP-1 in the control group, CUMS + siRNA group, and CUMS + PDTC group were significantly lower than those in the CUMS group. These cytokines were measured by ELISA according to the manufacturer's instructions. The agents (siRNA and PDTC) were given to mice 30 min before the stress exposure. Data are mean ± SEM (*n* = 15 per group). *P < 0. 05, **P < 0.01.

**Fig 7:**
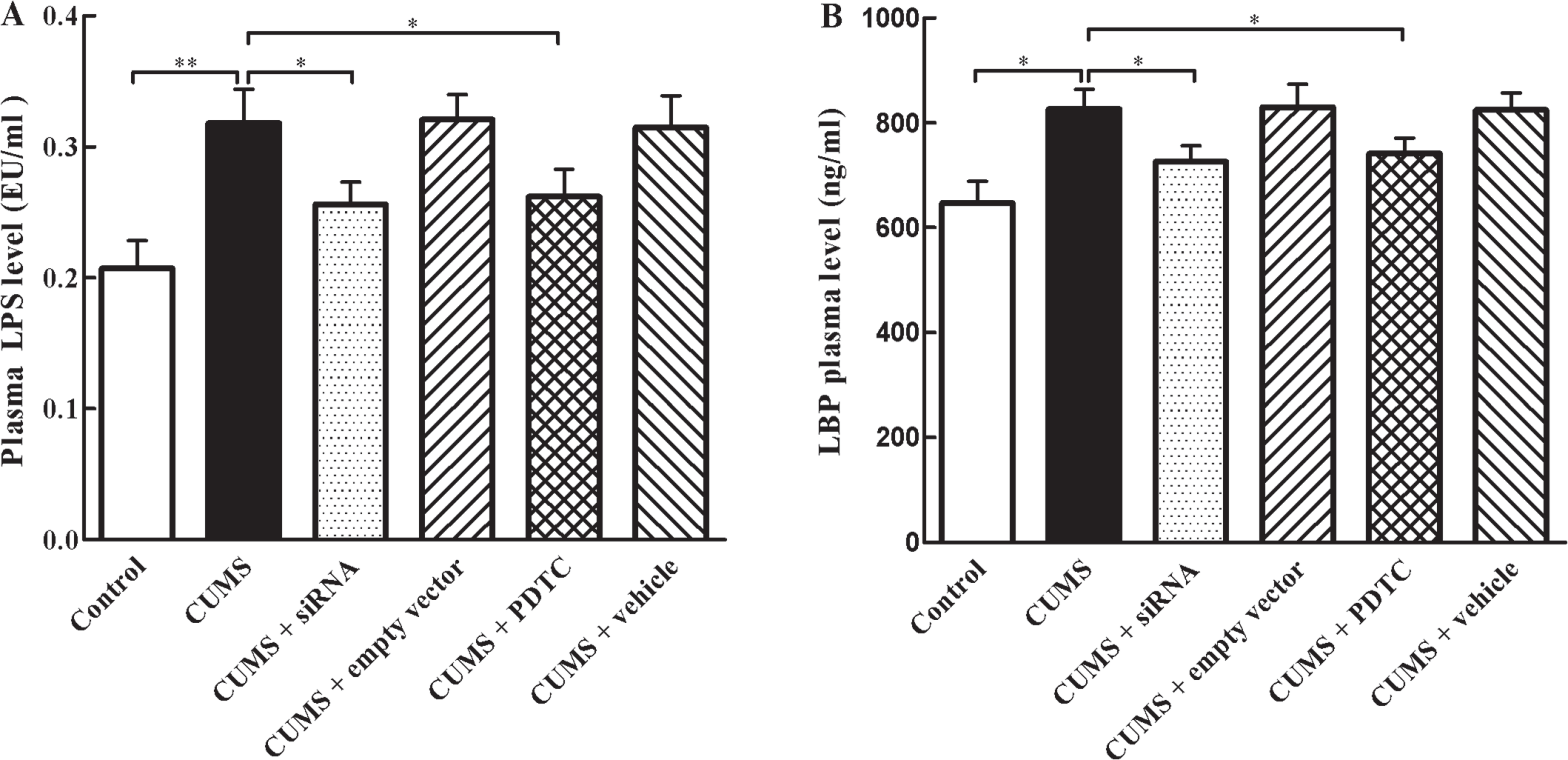
Effect of TLR4 siRNA and PDTC treatment on plasma LPS and LBP levels. (A) LPS and (B) LBP levels were determined by commercially available kits following the manufacturer’s instructions. The plasma levels of LPS and LBP in the control group, CUMS + siRNA group, and CUMS + PDTC group were significantly lower than those in the CUMS group. The agents (siRNA and PDTC) were given to mice 30 min before the stress exposure. Data are mean ± SEM (*n* = 15 per group). *P < 0. 05, **P < 0.01.

The activation of TLR4/NF-*κ*B signaling is known to promote numerous proinflammatory cytokines release. Considering the achieved results of TLR4/NF-*κ*B signaling was activated in the aorta of CUMS apoE^-/-^ mice, we measured the concentrations of circulating IL-1β, TNF-α and MCP-1 in plasma by ELISA analysis. As shown in Fig 6, consistent with the increased inflammatory response in the aortic wall, the plasma levels of IL-1β (Fig 6A, *P* < 0.01), TNF-α (Fig 6B, *P* < 0.05) and MCP-1 (Fig 6C, *P* < 0.05) were markedly elevated in the CUMS group compared to the control, respectively. As expected, the plasma proinflammatory cytokines IL-1β (Fig 6A), TNF-α (Fig 7B), and MCP-1 (Fig 6C) levels in CUMS + siRNA group, CUMS + PDTC group were significantly decreased, compared with CUMS group, respectively. While those proinflammatory cytokines levels were similar among CUMS + empty vector group, CUMS + vehicle group, and CMS group (*P* > 0.05). These data indicated that inhibition of TLR4/NF-*κ*B reduced circulating inflammatory cytokines IL-1β, TNF-α, and MCP-1 production in CUMS apoE^-/-^ mice.

### Inhibition of TLR4/NF-*κ*B reduces plasma LPS and LBP levels in CUMS apoE^-/-^ mice

LPS is a major ligand of TLR-4, whose activation switches on intracellular inflammatory signaling. In order to clarify the origin of the stress-induced activation of the TLR-4 pathway, plasma concentrations of LPS and LBP were evaluated. The results indicated that CUMS exposure caused an increase in both LPS and LBP plasma levels in apoE^-/-^ mice (Fig 7A and 7B). While those two proteins levels were significantly decreased in CUMS + siRNA group and CUMS + PDTC group compared with CUMS group, respectively.

## Discussion

In this study, we have investigated the role of TLR4/NF-*κ*B pathway in CUMS-induced atherosclerosis in apoE^-/-^ mice. Our results showed that CUMS exposure significantly increased the concentrations of corticosterone in plasma, indicating the presence of a systemic stress response. Next, we found that CUMS markedly aggravated the development of atherosclerosis, consistent with our and other lab's data [9–12]. Furthermore, we found that CUMS-induced atherogenesis can be interfered by inhibitions of TLR4 and NF-*κ*B. As our knowledge, this is the first evidence revealed that the activation of TLR4/NF-*κ*B pathway may be associated with CUMS-induced development of atherosclerosis.

Growing evidence has shown that TLR4 signaling-mediated immune response may play a critical role in the initiation and progression of atherosclerosis [20, 32–34]. Our previous study indicated that CUMS dramatically accelerated atherosclerosis and elevated TLR4 and NF-*κ*B in apoE^-/-^ mice [11]. Recent studies from other labs also revealed that TLR4 contributed to the inflammation induced by stress [23, 25, 35]. To address the pathophysiological roles of TLR4/NF-*κ*B signaling in the development of atherosclerosis induced by CUMS, we evaluated the influence of TLR4 knockdown and NF-*κ*B inhibition on the atherosclerosis in apoE^-/-^ mice. As expected, down-regulated TLR4 expression by Ad-TLR4 siRNA caused remarkable decreases in aortic atherosclerosis lesions of CUMS mice. The same trend was observed in the CUMS mice treated with NF-*κ*B antagonist PDTC. Interestingly, while no substantial changes in plasma corticosterone concentrations and lipid profile were observed in these mice, suggesting that the mechanisms of knockdown TLR4/NF-*κ*B pathway decreased CUMS-induced atherosclerosis in apoE^-/-^ mice may be independent of regulating lipid metabolism and corticosterone levels. Rozkva et al. reported that stressed-level corticosterone increased TLR4 expression in vitro cultured cells [36]. Consistent with other studies [25, 27], our data showed that CUMS-induced atherosclerosis might be associated with the inflammatory response caused by TLR4 ligands, such as oxidized low density lipoprotein (oxLDL), heat-shock proteins (HSPs) and LPS. Therefore, knockdown TLR4 expression attenuated CUMS-induced atherosclerosis maybe though lowering the downstream inflammatory cytokines expression in apoE^-/-^ mice.

Inflammation plays a crucial role in the development of atherosclerosis. Studies have shown that TLR4/NF-*κ*B signaling might be the important associated with inflammation and atherosclerosis [19–21, 31, 37]. NF-*κ*B is composed of two subunits, p65 (RelA) and p50. Inactive NF-*κ*B is sequestered in the cytoplasm, bound by members of the I*κ*B family of inhibitory proteins including I*κ*B-α. TLR4 can recognize various endogenous and extragenous ligands, which results in I*κ*B-α phosphorylation and subsequent degradation, nuclear translocation NF-*κ*B, and promoting the release of downstream pro-inflammatory cytokines and chemokines. The increased proinflammatory cytokines such as IL-1β and TNF-α are subclinical markers that predict atherosclerotic diseases [38]. Our previous study showed that elevated TLR4 expression and activated NF-*κ*B activity were observed in aortic atherosclerosis lesions of CUMS apoE^-/-^ mice [11]. To further reveal the mechanisms of reducing atherosclerosis lesions by blocking TLR4/NF-*κ*B, we investigated the effects of Ad-TLR4 siRNA and NF-*κ*B antagonist PDTC on inflammatory cytokines such IL-1β, MCP-1, and TNF-α. Present results indicated that CUMS increased the protein expressions of TLR4, intra-nuclear NF-*κ*B p65 and decreased I*κ*B-α expression compared with control group. And importantly, our data demonstrated that TLR4 siRNA and PDTC treatment significantly reduced TLR4 and NF-*κ*B expression and enhanced the I*κ*B-α in the arteries of CUMS apoE^-/-^ mice. Accordingly, the levels of IL-1β, MCP-1, and TNF-α, which are downstream signaling molecules of TLR4/NF-*κ*B pathway, were markedly decreased in these mice. These results are in agreement with other studies in which proinflammatory cytokines levels much lowered in TLR4-decicient or knockdown mice after chronic stress [24, 26, 28]. The reduced inflammatory response found in TLR4 siRNA and PDTC treated mice can be explained by the fact that these mice have a defective response to TLR4 ligands. Since TLR4 activates NF-*κ*B pathways to produce the proinflammatory cytokines, the signaling linked to NF-*κ*B in TLR4 siRNA treatment mice are likely to be inhibited. Furthermore, NF-*κ*B is a critical downstream target of TLR4 signaling [39]. Thus, PDTC could block TLR4 signaling and prevent the increases in IL-1β, MCP-1, and TNF-α. Given that TNF-α can elevate the expression of TLR4, blocking NF-*κ*B decreased TLR4 expression through reducing TNF-α production. Interestingly, these results did not contribute to a different response to stress, because there were no significant changes in plasma concentrations of corticosterone between CUMS and TLR4 siRNA, and PDTC treatment CUMS apoE^-/-^ mice.

A recent study showed that chronic stress accelerated haemopoietic stem cell proliferation and enhanced neutrophil and monocyte production, resulting in extensive release of inflammatory leukocytes into the circulation [40]. TLR4 was expressed on these inflammatory leukocytes. Once activated by TLR4 ligands, these leukocytes promote expression and synthesis of the inflammatory cytokines IL-1β, IL-6, and TNF-α. Furthermore, there was evidence indicated that TLR4-promoted neutrophil survival depended upon signaling via NF-*κ*B. These above data suggested that TLR4 siRNA and PDTC treatment decreased plasma levels of IL-1β, MCP-1, and TNF-α in CUMS mice maybe through reducing leukocyte numbers in the blood.

It is necessary to consider the potential ligands of TLR4 under stress that may contribute to inflammation and atherosclerosis in CUMS apoE^-/-^ mice. Recent studies have shown that the exposure to chronic stress may induce endogenous ligands of TLR4 release from activated endothelium and macrophages, such as heat shock protein 70, and ox-LDL [11, 27]. Furthermore, exposure to chronic stress may damage the intestinal barrier, making it leaky and enhancing the LPS concentrations in blood, an important ligand of TLR4 [23]. Once TLR4 activated by these ligands, it would initiate intracellular signal transduction, thereby activating NF-*κ*B, which leads to increase in expression of the inflammatory cytokines such as IL-1β, MCP-1, and TNF-α. These cytokines can cause endothelial dysfunction, promote monocytes infiltration into the vessel walls, and sustain inflammation. Our present results showed that TLR4 siRNA and PDTC treatment can decrease plasma LPS levels in CUMS apoE^-/-^ mice, which may further reduce the activation of TLR4 pathway-induced inflammation.

As the limitation of our study, although we have shown the proatherogenic actions of TLR4/NF-*κ*B pathway in CUMS apoE^-/-^ mice, we did not measured the effect of CUMS on TLR4 expression on endothelial cells, macrophages, and vascular smooth muscular cells within vessel wall. TLR4 is widely expressed on different cell types in vessel wall known to be involved in the pathogenesis of atherosclerosis. TLR4 activation by its ligands elicits signal transduction to induce the inflammatory response, which results in initiation and progression of atherosclerosis. Further studies are needed to explore which TLR4 is a critical to inhibit atherosclerosis-induced by CUMS. In addition, it is possible that some mechanisms other than the activation of NF-*κ*B-mediated signals were also involved in CUMS-induced atherosclerosis.

In summary, our studies demonstrate that TLR4/NF-κB pathway is involved in CUMS-induced the development of atherosclerosis. CUMS response may activate TLR4, thereby increasing proinflammatory cytokines release through the NF-*κ*B pathway to promote inflammation response, thus contributing to the development of atherosclerosis. It may provide a potential therapeutic target for prevention and treatment this disease.
